# Variation in Interleukin-4, -6, and -10 in Mastitis Milk: Associations with Infections, Pathogens, Somatic Cell Counts, and Oxidative Stress

**DOI:** 10.3390/vetsci11080350

**Published:** 2024-08-02

**Authors:** Wasana Chaisri, Montira Intanon, Duanghathai Saipinta, Anyaphat Srithanasuwan, Noppason Pangprasit, Weerin Jaraja, Areerat Chuasakhonwilai, Witaya Suriyasathaporn

**Affiliations:** 1 Faculty of Veterinary Medicine, Chiang Mai University, Chiang Mai 50100, Thailand; wasana.ch@cmu.ac.th (W.C.); montira.intanon@cmu.ac.th (M.I.); duanghathai.s@cmu.ac.th (D.S.); weerin.kim@gmail.com (W.J.); areeratc.yok@gmail.com (A.C.); 2 Research Center of Producing and Development of Products and Innovations for Animal Health and Production, Chiang Mai University, Chiang Mai 50100, Thailand; 3 Department of Animal Sciences, Wageningen University, 6700 AH Wageningen, The Netherlands; numwan.sw@gmail.com; 4 Akkhraratchakumari Veterinary College, Walailak University, Nakhon Si Thammarat 80160, Thailand; p.noppason@gmail.com; 5 Cambodia Campus, Asian Satellite Campuses Institute, Nagoya University, Nagoya 464-8601, Japan

**Keywords:** concomitant infection, interleukin, mastitis, streptococci, staphylococci, MDA

## Abstract

**Simple Summary:**

This study examines the correlation between interleukin-4 (IL-4), interleukin-6 (IL-6), and interleukin-10 (IL-10) levels in mastitis milk and concurrent infections, bacterial pathogens, somatic cell counts (SCCs), and malondialdehyde (MDA), an oxidative stress marker. The bacterial identification of all mastitis quarters from five smallholder dairy farms was conducted using MALDI-TOF mass spectrometry. The samples were collected aseptically before morning milking and re-sampled before afternoon milking. The concomitant infection between streptococci and coagulase-negative staphylococci (CNS) was selected, and the samples were randomly selected as a controlled single infection. The results indicated a substantial positive correlation between SCC and IL-4, whereas interleukin-6 exhibited a negative trend. CNS exhibited the highest levels of IL-4, which were substantially higher than those of mixed bacteria and of none. A quarter were infected with streptococcal bacteria, and mixed bacteria exhibited the maximum IL-6 levels.

**Abstract:**

Poor mastitis control favors intramammary infection (IMI), which always involves CNS. This study aimed to determine the relationships of IL-4, IL-6, and IL-10 in mastitis milk with concurrent infection, bacterial pathogens, SCC, and MDA, an oxidative stress marker. All mastitis quarters from five smallholder dairy farms were sampled aseptically before morning milking and again before afternoon milking for bacteriological identification using MALDI-TOF mass spectrometry. The samples with the concomitant infection between streptococci and CNS and their pairs of another sample from the quarters were selected. In addition, samples were randomly chosen to have a controlled single infection. IL-4, IL-6, and IL-10 were measured with ELISA kits. MDA was measured using HPLC, while SCC was measured using Fossomatic™ FC. The results from a repeated measure analysis showed that IL-4 positively correlated with SCC, while IL-6 showed a negative trend. IL-4 levels were highest in CNS infections and significantly higher than in non-infected or mixed infections (*p* < 0.05). The IL-6 level of the mixed bacteria was highest and showed a different trend from non-infection, and the quarter was infected with streptococcal bacteria. In conclusion, from a single infection, the streptococci and CNS quarter showed varied immune responses, including trendily higher IL-6 and IL-4.

## 1. Introduction

Mastitis causes significant economic losses and impacts animal welfare due to reduced milk quality and increased antibiotic use. Mastitis incidence is influenced by factors such as the virulence and quantity of invading microbes, the efficiency of the udder defense mechanism, and environmental risk factors. Poor mastitis control programs favor intramammary infection (IMI) from heterogeneous bacteria, always causing a concomitant infection [1], and CNS was the most common bacteria causing the concomitant infection [2]. Bacteria likely use competitive mechanisms to survive and shape communities in any environment and host organisms [3]. The results of bacterial competition always show a single dominant mastitis pathogen in most mastitis studies [4]. In their 2013 study, Keane et al. found that the bacteria causing mastitis, as identified by PCR, were often multiple mastitis pathogens [5].

Once bacteria invade the udder, the defense mechanism tries to resolve the IMI immediately [4,6]. The recognition of invading microbes signals the secretion of cytokines and chemokines, including interleukin-1 (IL-1β), IL-4, IL-6, IL-8, and IL-10 involved in recruiting phagocytes to the site of infection and activating pathogen-killing mechanisms [7,8], and subsequently recruits a huge amount of leukocytes into the infected quarter, causing an increase in SCC. IL-6 is also an important cytokine that could detect early stages of subclinical mastitis [9]. The phagocytes kill bacteria using oxygen-dependent and oxygen-independent processes using superoxide, which is converted to other reactive oxygen species (ROS), resulting in oxidative stress. Malondialdehyde (MDA), an indicator of oxidative stress, was related to SCC in milk [10].

Successful eradication leads to the destruction of bacteria, and the recruitment of neutrophils from the acute phase terminates. However, in case of unsuccessful elimination, the IMI can proceed into an aggressive immune response to clinical mastitis or a less aggressive immune response, leading to chronic subclinical mastitis [11]. Factors such as bacterial species, pathogen load, and host defense efficiency influence the unsuccessful elimination of bacterial infections. For instance, *S. agalactiae*, a contagious mastitis pathogen, exhibits a significantly low spontaneous cure rate and prolonged duration of intramammary infection [12], while CNS could cause a short spontaneous cure within one week after IMI [13]. Previous research has shown that varying infections elicit varying immune responses [7,8,9,10]. Interleukin, cytokine, and MDA levels in mastitis-infected quarters might be related to a variety of factors, including mixed infections and bacterial species, which may influence spontaneous recovery. Although the udder’s defense mechanism is well understood, the immune response to concurrent infections is still limited. Concentrations of cytokines in mastitis-infected quarters could be related to various factors, including concomitant infections, bacterial species, and MDA. Therefore, this study aimed to determine the relationships of IL-4, IL-6, and IL-10 in mastitis milk with concurrent infections, bacterial pathogens, SCC, and oxidative stress.

## 2. Materials and Methods

### 2.1. Ethics Approval

The research protocol was reviewed and approved by the Animal Care and Use Committee (FVM-ACUC) of the Faculty of Veterinary Medicine, Chiang Mai University (Protocol # R19/2565).

### 2.2. Farms and Animals

This study used 183 cows from five smallholder dairy farms with high-bulk milk SCC > 800 × 10^3^ cells/mL in the Mae-On sub-district, Chiang Mai Province, from October to December 2022. The farms participated in the Herd Health Management Program, Chiang Mai Artificial Insemination and Biotechnology Research Center, Department of Livestock Development, Ministry of Agriculture and Cooperatives, Thailand. The program included a mastitis control program by checking the SCC of the composite milk of all cows. In all farms, cows were housed in tied-stall barns, fed postharvest corn stem and rice straw ad-lib, and in concentrates according to their milk production. The vast majority of cows were cross-bred Holstein–Friesian. Bucket-type milking machines were used on all farms. The vacuum level ranged from 45 to 50 kPa, the pulsation rate ranged from 58 to 60 beats per minute, and the pulsation ratio was 60:40.

Cows with the composite SCC > 200 × 10^3^ cells/mL were selected and tested before the morning milking for either subclinical mastitis, using the California mastitis test at the score ≥1, or clinical mastitis. All affected quarters were sampled aseptically and re-sampled before the afternoon milking for bacteriological identification and measurement of SCC. All quarter milk samples, at least 1 out of 2 samples showing the concomitant or mixed infections, were selected for further analyses of IL-4, IL-6, IL-10, and MDA. In addition, the samples were simply, randomly chosen to have a controlled single infection.

### 2.3. Bacterial Identification

Mastitis-associated pathogens were cultured according to the National Mastitis Council guidelines. Subsequently, the colonies were identified by MALDI-TOF mass spectrometry (Shimadzu Biotech, Kyoto, Japan), a technique used for identifying bacteria based on protein profiles to verify the distinction between genera.

### 2.4. SCC Analysis

According to the manufacturer’s instructions, somatic cells in milk samples were analyzed using Fossomatic™ FC (Foss, Hilleroed, Denmark).

### 2.5. Cytokines Analysis

Commercially available ELISA kits (Abbexa LLD., Houston, TX, USA) were used to measure IL-4, IL-6, and IL-10 levels in milk samples. All testing was carried out according to the manufacturer’s instructions. The cytokine concentrations in milk were calculated using a standard curve with seven concentrations ranging from 15.6 to 1000 pg/mL. The measurement was duplicated.

### 2.6. MDA Analysis

MDA was extracted according to Fenaille et al. [14]. The extracted samples were analyzed for MDA using high-performance liquid chromatography (HPLC) (Shimadzu Biotech, Kyoto, Japan), with a UV detector with an absorbance of 532 nm. The column employed was the Inersil ODS-3 C18 (5 µm, 250 mm × 4.6 mm). The study was conducted isocratically, with a mobile phase of phosphate buffer (50 mM, pH 6) and methanol in a 60:40 ratio. The flow rate was 1 mL/min, with an injection volume of 20 µL.

### 2.7. Statistical Analysis

All data were described as percentages and means. Data on IL-4, IL-6, IL-10, SCC, and MDA concentrations were tested for normality using Shapiro–Wilk tests. Non-normal data were log-transformed. Due to the duplicated measurement of cytokines of samples from the same quarter before morning (BM) and before afternoon milking (BA), a repeated measured analysis using a single mixed linear model was separately created for IL-4, IL-6, and IL-10 as outcome variables. The independent variables, taken as the fixed effects, included the number of IMI as single or mixed (as >2 IMI in the same quarter/time); milking time as BM or BA; number of infected bacteria as none, single, and mixed; infected bacteria as none, streptococci (*S. agalactiae*, *S. dysgalactiae*, *S. uberis*), CNS (*S. chromogenes*, *S. haemolyticus*), and mixed; SCC; and MDA. SCC and MDA were taken as continuous variables; the rest were categorical. Data on the cow, quarter identifications, and measurement times were defined as repeated variables. The least-square means were calculated for each level of the category variables, and the graph was displayed using the least-square mean values. The significant differences were determined at *p* < 0.05, and the trend was defined at *p* < 0.10.

## 3. Results

### 3.1. Bacterial Isolates

From 174 CMT-positive quarters, 134 and 124 were confirmed to show bacterial identification at BM and BA, respectively. A total of eight quarters were found to be mixed infections between streptococci and CNS, including seven (5.2%) and one (0.8%) for morning and afternoon, respectively. The control samples of the same quarter were collected from another milking time (n = 8). Samples from nine quarters with none or one infection collected were reserved as samples from control quarters. One sample was excluded due to its infection with Candida tropicalis. The mixed infections included *S. agalactiae* with *S. chromogenes* (n = 1), *S. agalactiae* with *S. argenteus* (n = 1), *S. uberis* with *S. chromogenes* (n = 2), and *S. uberis* with *S. haemolyticus* (n = 3). The single infections included *S. agalactiae* (n = 4), *S. dysgalactiae* (n = 5), *S. uberis* (n = 12), and *S. chromogenes* (n = 3), and 2 samples had no infection. All bacteria species were re-coded in the bacterial group variable as none (n = 2), streptococci (n = 28), or CNS (n = 10).

### 3.2. The Concentrations of IL-4, IL-6, IL-10, SCC, and MDA

Table 1 shows that the mean levels of IL-4, IL-6, IL-10, SCC, and MDA were 3111, 937.7, 43.53 pg/mL, 1403 × 10^3^ cells/mL, and 20.7 nmol/mL, respectively. Except for IL-6, all other variables were taken as logarithms for their non-normal data, but only IL-4 could improve their normal distribution and be used for further analysis.

### 3.3. Relationship between Interleukin, SCC, and Infection Type

**Figure 1:**
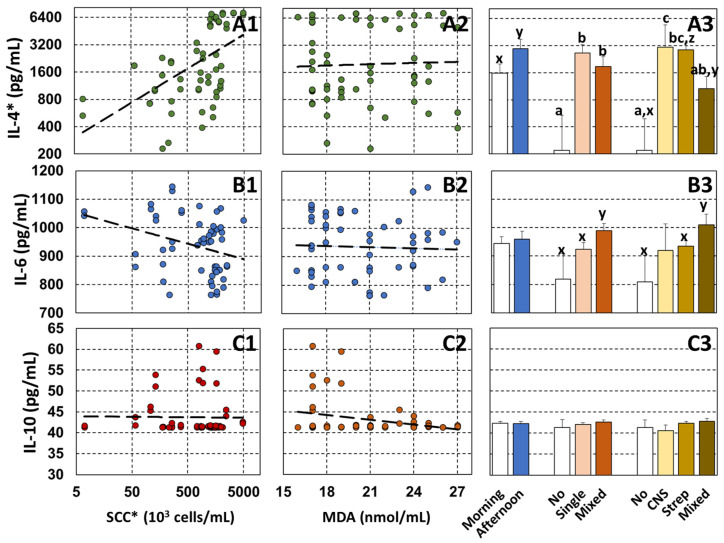
Relationship of somatic cell count (SCC) and malondialdehyde (MDA), sampling times, number of infections, and infected bacteria on interleukin-4 (IL-4), IL-6, and IL-10, respectively. (**A1**) Relationship of IL-4 and SCC. (**A2**) Relationship of IL-4 and MDA. (**A3**) Relationship of IL-4 and sampling times, number of infections, and infected bacteria. (**B1**) Relationship of IL-6 and SCC. (**B2**) Relationship of IL-6 and MDA. (**B3**) Relationship of IL-6 and sampling times, number of infections, and infected bacteria. (**C1**) Relationship of IL-10 and SCC. (**C2**) Relationship of IL-10 and MDA. (**C3**) Relationship of IL-10 and sampling times, number of infections, and infected bacteria. * Indicated logarithm-transformation scale for SCC and IL-4. a,b,c and x,y,z letter differences indicate significant differences at *p* < 0.05 and *p* < 0.1, respectively. No (none infection); CNS (coagulase-negative staphylococci) included *S. chromogenes* and *S. haemolyticus*; Strep (streptococcal bacteria) included *S. agalactiae*, *S. dysgalactiae*, and *S. uberis*. The least-square means of IL-4, IL-6, and IL-10 of the model with the specified variables were used to describe in (**A3**,**B3**,**C3**).

The relationship between SCC and MDA, milking times, number of infections, and infected bacteria on IL-4, IL-6, and IL-10 are shown in Figure 1. No relationships were found between MDA and all interleukins (Figure 1(A2,B2,C2)). Also, no factor was significantly related to IL-10. Figure 1(A1) shows a significant positive relation between IL-4 and SCC, while IL-6 had a negative trend relation to SCC (Figure 1(B1)). The results from statistical analyses show significant relationships between IL-4 and SCC (b = 0.516, *p* < 0.01), and IL-6 and SCC (b = -24.28, *p* = 0.14). For the milking time, a least-square mean of IL-4 from BM (1571.6 pg/mL) showed a trend in difference from that of BA (2941.9 pg/mL) at *p* = 0.07 (Figure 1(A3)). The mean of IL-4 non-infection (220.3 ± 316.0 pg/mL) was lower than those of the single infection (2635.9 ± 634.9 pg/mL) and the mixed infection (1875.6 ± 525.0 pg/mL) at *p* < 0.05. IL-4 was the highest in CNS at 3059.2 ± 2365.9 pg/mL and significantly higher than the none at 219.5 ± 274.1 pg/mL and the mixed bacteria at 1063.8 ± 381.1 pg/mL. No difference was found between CNS and streptococci (Figure 1(A3)). In contrast, the IL-6 level of the mixed infected number (988.9 ± 26.3 pg/mL) was higher than those of the none (819.0 ± 91.0 pg/mL) and the single infection (924.0 ± 23.6 pg/mL) at *p* < 0.1 (Figure 1(B3)). For the infected bacteria, IL-6 of the mixed bacteria was highest and showed a trend in difference from the none and the quarter infected with streptococcal bacteria.

## 4. Discussion

Without infection, the serum IL-6 level is very low, but it increases rapidly and sharply during the early stage of bacterial infection [15,16], while IL-10 is a potent anti-inflammatory cytokine that works to prevent inflammatory and autoimmune pathologies [17]. Likewise, IL-4 is important in leukocyte survival under physiological and pathological conditions [18]. Many studies have compared the acute-phase response after infection in healthy cows to that of infected cows. However, this study’s infected stage might indicate a majority of persistent mastitis, as is often the case in high-bulk SCC farms [12]. In addition, the present study aimed to differentiate the mechanisms related to single and mixed infections. The heterogeneous bacteria IMI always caused mixed and concomitant infection [1], especially with CNS [2]. It is important to consider all further applications of this study carefully.

There are limitations in the number of publications on IL-4, IL-6, and IL-10 in milk. In 2017, average concentrations of IL-4, IL-6, and IL-10 in subclinical mastitis milk with CNS were reported as lower than the values from this study [19]. In addition, Hagiwara and colleagues [18] also reported lower IL-6 levels in mastitis milk with a single infection. In contrast, the average IL-4 level for non-infection in this study at 219.5 ± 274.1 pg/mL was lower than the median values at 670.2 pg/mL reported by Bochniarz et al. [19]. The concentration of IL-10 in this study was in the ranges of both previous studies. The higher concentrations of IL-4 and IL-6 in the infected quarters in this study might be due to chronic mastitis in the farms with mastitis control problems. For MDA, the values reported here were in the range of our previous studies [10]. No relationships were found between MDA and any interleukins (Figure 1(A2,B2,C2)). Upon asthmatic immunopathology, plasma levels of MDA were significantly (*p* < 0.001) enhanced in asthmatics and were significantly related to IL-6 [20], while a weak positive correlation between IL-6 and MDA was found in HIV-infected patients with or at risk of Kaposi’s disease [21]. The differences in statistical methods, diseases, and the narrow range at 16 and 27 nmol/mL of MDA might have resulted in the lack of association between MDA and cytokines in this study. Also, no factor was significantly related to IL-10 levels (Figure 1(C1–C3)). This might be caused by most samples being chronic IMI samples. However, because of the limited sample size we randomly selected, larger-scale studies are required to better understand the relationship between milk interleukins and mastitis in dairy cows.

A significant positive relation between IL-4 and SCC was found (Figure 1(A1)), while IL-6 had a negative trend relation to SCC. SCC indicates udder inflammation and an immune response due to bacterial infection. IL-4 can stimulate the proliferation and activation of immune cells, including leukocytes, which are major components of SCC that promote tissue repair in ongoing inflammation. In general mastitis cases, immunoreactivity was more pronounced for IL-4, IL-6, IL-12, IL-13, IL-17A, TNF-α, and IFN-γ. Subclinical and clinical mastitis data demonstrate inflammatory responses to intramammary infection driven by IL-1α, IL-4, and IL-17A [10]. In contrast, IL-4 gene expression and levels in milk were lower in mastitis animals compared to healthy animals [19,22,23]. IL-4 responds to several stages of inflammation, including suppressing the initial inflammatory response to injury and regulating the modification of the extracellular matrix for effective repairs [24]. Disturbances in the levels of inflammatory cytokines are responsible for the development of chronic inflammation. IL-4 is related to continuing or chronic inflammation, as well as wound healing, by activating endothelial cells and fibroblasts. Previous research has shown an association between IL-4 and chronic inflammatory disorders [25]. In this study, the possible chronic mastitis cases might be related to several steps, including inflammation and tissue repair, causing the differences in the relationship between IL-4 and SCC. Chronic mastitis, which is characterized by persistent inflammation of the mammary gland, frequently results in higher IL-4 levels as part of the immune system’s attempt to fight the infection, which is directly increased in SCC. Therefore, the overexpression of IL-4 in this study might also be from chronic mastitis.

This study (Figure 1(A3)) shows similar levels of IL-4 in streptococci and CNS. This was supported by the finding of no difference in IL-4 between mastitis milk with *S. aureus* and *S. agalactiae* [23]. This is the first report on the differences in responses of IL-4 and IL-6 to concomitant infection. Concomitant CNS in the streptococci mastitis quarter had the highest IL-6 level but the lowest IL-4 level. In concomitant infections, the burden of one or both infectious agents may be increased, one or both may be suppressed, or one may be increased and the other may suppress immune function [26,27]. The efficiency of the immune system’s defense against pathogens, but also the severity of its attacks against itself after tolerance breakdown, are largely affected by intrinsic (for example, genetics) and extrinsic factors (for example, environmental cues or exposome), including exposure to pathogenic and commensal microorganisms. Increased IL-6 in the mixed infection might indicate a higher number of pathogens and, subsequently, an increase in the acute-phase response. When dairy cows have new bacterial infections over the existing mastitis, many inflammatory and immunologic cells in the body can rapidly express the IL-6 gene and produce IL-6 [28,29].

## 5. Conclusions

Concomitant infection, especially from CNS, occurs frequently on mastitis quarters in poor mastitis-controlled program farms. This study found significant differences in IL-4 and IL-6 levels in mastitis milk depending on the type of bacterial infection. This helps veterinarians to understand the immune response mechanism of the concomitant infection, causing spontaneous cure of the concomitant infection. Future studies should explore the potential of using cytokine profiles for the early detection and management of mastitis.

## Figures and Tables

**Table 1:** The concentrations of IL-4, IL-6, IL-10, SCC, and MDA in mastitis milk.

	IL-4 (pg/mL)	IL-6 (pg/mL)	IL-10 (pg/mL)	SCC (Cells/mL)	MDA (nmol/mL)
Range	230–7068	764.9–114.3	41.31–60.87	7–6728 × 10^3^	16–27
Mean	3111	937.7	43.53	1403 × 10^3^	20.7

## Data Availability

All data are shown in the manuscript.
